# Clinical Development and Evaluation of a Multi-Component Dissolving Microneedle Patch for Skin Pigmentation Disorders

**DOI:** 10.3390/polym15153296

**Published:** 2023-08-04

**Authors:** Chenxin Yan, Mengzhen Xing, Suohui Zhang, Yunhua Gao

**Affiliations:** 1 Key Laboratory of Photochemical Conversion and Optoelectronic Materials, Technical Institute of Physics and Chemistry, Chinese Academy of Sciences, Beijing 100190, China; yanchenxin20@mails.ucas.ac.cn; 2 University of Chinese Academy of Sciences, Beijing 100049, China; 3 Key Laboratory of New Material Research Institute, Department of Acupuncture-Moxibustion and Tuina, Shandong University of Traditional Chinese Medicine, Jinan 250355, China; mengzhen@mail.ipc.ac.cn; 4 Beijing CAS Microneedle Technology Ltd., Beijing 102609, China

**Keywords:** glabridin, 3-O-Ethyl-L-ascorbic acid, tranexamic acid, microneedles, pigmentation, multifunctional drug

## Abstract

Excessive melanin deposition in the skin leads to various skin pigmentation diseases, such as chloasma and age spots. The deposition is induced by several factors, including tyrosinase activities and ultraviolet-induced oxidative stress. Herein, we propose a multi-component, multi-pathway drug combination, with glabridin, 3-O-ethyl-L-ascorbic acid, and tranexamic acid employed as, respectively, a tyrosinase inhibitor, an antioxidant, and a melanin transmission inhibitor. Considering the poor skin permeability associated with topical application, dissolving microneedles (MNs) prepared with hyaluronic acid/poly(vinyl alcohol)/poly(vinylpyrrolidone) were developed to load the drug combination. The drug-loaded microneedles (DMNs) presented outstanding skin insertion, dissolution, and drug delivery properties. In vitro experiments confirmed that DMNs loaded with active ingredients had significant antioxidant and inhibitory effects on tyrosinase activity. Furthermore, the production of melanin both in melanoma cells (B16-F10) and in zebrafish was directly reduced after using DMNs. Clinical studies demonstrated the DMNs’ safety and showed that they have the ability to effectively reduce chloasma and age spots. This study indicated that a complex DMN based on a multifunctional combination is a valuable depigmentation product worthy of clinical application.

## 1. Introduction

The normal deposition of melanin in the basal layer of the skin results in the pigmentation of human skin and hair. Aside from conferring protection against ultraviolet (UV) rays, excessive melanin deposition leads to melasma [1] and age spots [2]. Specifically, melasma occurs because of inducing factors such as UV radiation, hormone levels, and family history [1,3]. However, the exact mechanism of age spot formation is currently unclear, and it is widely believed that UV radiation is the main cause of its formation [4].

Melanin production is governed by the rate-limiting enzyme tyrosinase [5]. UV radiation indirectly stimulates tyrosinase, thus increasing melanin production. Recently, several tyrosinase inhibitors intended for skin whitening have been developed, including hydroquinone [6], kojic acid [7], arbutin [8], and aloesin [9]. However, these agents can cause significant side effects, including skin irritation, inflammation, and potential teratogenicity [10]. Furthermore, UV radiation can induce oxidative stress [11], which leads to the formation of oxidants such as nitric oxide [12] and which indirectly stimulates skin pigmentation. Therefore, certain antioxidants can decrease melanin production, such as glutathione [13], ascorbic acid (ASA) [14], and ferulic acid [15]. Recently, researchers have combined tyrosinase inhibitors and antioxidants, to treat pigmented lesions. For instance, Yu et al. [16] showed that combining the tyrosinase inhibitor azelaic acid with the antioxidant taurine significantly improved melanin inhibition compared to using azelaic acid alone.

**Figure 1:**
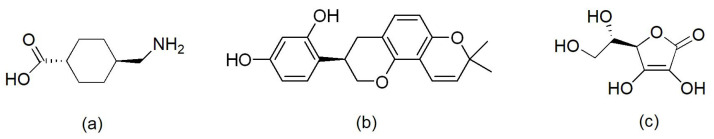
The chemical structural formula of (**a**) TXA; (**b**) GLA; and (**c**) EAA.

Tranexamic acid (TXA) (seen in Figure 1a), which is a derivative of lysine, was initially proven to be an anti-fibrinolytic agent [17]. It was discovered that TXA has great whitening effects, and it is commonly used to treat melasma through oral administration, injection, or local application [18,19]. However, the systemic administration of TXA usually leads to adverse effects in patients, such as gastrointestinal discomfort and skin rash [20]. Glabridin (GLA) (seen in Figure 1b), which comes from the flavonoid group of compounds in the root of glycyrrhiza glabra, is an excellent tyrosinase inhibitor. It can effectively reduce the transformation of tyrosine to melanin in melanocytes [21,22,23]. Additionally, 3-O-Ethyl-L-ascorbic acid (EAA) (seen in Figure 1c), a derivative of vitamin C (VC), has better stability and hydrophilic–lipophilic balance than VC [24]. Simultaneously, EAA retains the apparent antioxidant effects of VC. The combined use of three effective components is expected to ameliorate stubborn, hard-to-treat skin hyperpigmentation, lengthy treatment, and high recurrence rates. However, because of the protective barrier function of the stratum corneum, drug absorption through the skin is challenging. Therefore, the key to the drug’s intended efficacy lies in achieving efficient transdermal drug delivery.

Microneedles (MNs) are a recent drug delivery innovation [25]. These needle tips, measuring several hundred micrometers in length, can create micrometer-sized pores on the skin, allowing drugs to bypass the skin barrier and to reach the site of the disease. Their small size ensures that they do not come into contact with blood vessels and nerves, which improves patient compliance and comfort, while reducing usage burden. The use of hyaluronic acid (HA) as a matrix for MNs is preferred, due to its excellent biocompatibility. The hyaluronic acid microneedle delivery system has been successfully applied to transport a variety of drugs, including vaccines [26], exenatide [27], proteins [28], and anesthetics [29]. To fabricate microneedle arrays, a mixture of three polymers with biocompatibility and flexibility was used: polyvinyl alcohol (PVA), polyvinyl pyrrolidone (PVP) [30], and hyaluronic acid (HA).

In this study, we employed MNs as drug delivery carriers to combine GLA, TXA, and EAA, aiming to synergistically inhibit melanin production from multiple perspectives. Through performing a series of experiments—including in vitro antioxidant assays, tyrosinase inhibition assays, melanin generation inhibition evaluations, zebrafish assays, and clinical trials—we examined the efficacy of the newly developed drug-loaded microneedles (DMNs). Moreover, the effectiveness of DMNs in decolorization was confirmed by in vitro and ex vivo experiments.

## 2. Materials and Methods

### 2.1. Materials

Tranexamic acid and Glabridin were purchased from Linkebe Technology Co., Ltd. (Hangzhou, China). 3-O-Ethyl-L-ascorbic acid was procured from Dezhou Anglida Biotechnology Co., Ltd. (Dezhou, China). The hyaluronic acid (HA, Mw: 240,000) was procured from Bloomage Biotech (Beijing, China). Poly(vinylpyrrolidone) (PVP) was bought from Boai Nky Pharma Co., Ltd. (Beijing, China). Poly(vinyl alcohol) (PVA) was purchased from Alpha Hi-Tech Pharm Co., Ltd. (Pingxiang, China). The 2-Phenyl-4,4,5,5-tetramethylimidazoline-1-oxyl-3-oxide (PTIO) was bought from Santa Cruz Biotechnology, Inc. (Santa Cruz, CA, USA), while 1,1-diphenyl-2-picrylhydrazyl (DPPH) was bought from Aladdin Biochemical Technology Co., Ltd. (Shanghai, China), and L-DOPA from Sigma-Aldrich (St. Louis, MO, USA). Dulbecco’s Modified Eagle’s Medium (DMEM low glucose), RPMI 1640 medium, fetal bovine serum (FBS), phosphate-buffered saline (pH = 7.0–7.4), penicillin–streptomycin (P/S), and 0.25% trypsin (1×) were procured from Gibco (Rockville, MD, USA). The Cell Counting Kit-8 assay kit was provided by Dojindo (Kumamoto Prefecture, Japan). Non-tissue/cell lysate was ordered from Solarbio Tech Co., Ltd. (Beijing, China), while RIPA buffer was bought from Beyotime Biotechnology (Shanghai, China). Sodium hydroxide and dimethyl sulfoxide were obtained from InnoChem (Beijing, China) and Beijing Chemical Works (Beijing, China), respectively. PBS (50 mM, pH = 6.8) was purchased from Regen Biotechnology Co., Ltd. (Beijing, China).

Sprague Dawley (SD) rats (male, 8 weeks old, 220 ± 20 g) were obtained from SPF Biotech Co., Ltd. (Beijing, China). Procedures for animal studies were approved by the Institutional Animal Care and Utilisation Committee of the Technical Institute of Physics and Chemistry, CAS (approval number, LHDW-23025 and IACUCIPC-23025). The animal experiments followed the Guide for the Care and Use of Laboratory Animals (Eighth Edition, 2011). B16-F10 cells were purchased from Shanghai Enzyme Research Biotechnology Co., Ltd. (Shanghai, China). The wild-type AB strain of zebrafish was obtained from China Zebrafish Resource Center (Wuhan, China), and their breeding and propagation followed the requirements of the international AAALAC certification (certification number: 001458). The animal experimentation procedures were executed according to The Standard Operating Procedures for Evaluating the Whitening Efficacy of Zebrafish.

### 2.2. Volunteers

For the safety evaluation of DMNs, we recruited 30 participants, comprising 6 males and 24 females, aged between 20 and 58 years old. To assess the efficacy of DMNs in reducing facial pigmentation, 6 eligible female participants, aged from 18 to 60 years old, with noticeable facial pigmentation were chosen based on a predetermined set of criteria. The recruitment of volunteers was carried out following the medical and ethical principles outlined in the Helsinki Declaration. Before participation, all volunteers had to provide their informed consent to be involved in the study by signing a written consent form. All aspects of the study, including but not limited to conducting the experiment, analyzing data, and compiling the report, adhered to the principles of Good Clinical Practice.

### 2.3. Preparation of DMNs

**Figure 2:**
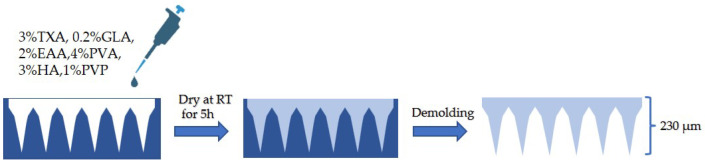
The manufacturing process of DMNs.

HA, PVA, and PVP were chosen as the primary matrix for MNs fabrication due to their outstanding biocompatibility [31]. When preparing the drug-containing solution, 3% TXA, 0.2% GLA, and 2% EAA were dissolved in deionized water. The pH of the solution was then adjusted to 5.5–6 and filtered through a 220 nm filter. PVA was dissolved in deionized water at 80 °C while HA and PVP were dissolved at room temperature. The three polymers were then mixed to form a homogeneous solution containing 4% PVA, 3% HA, and 1% PVP. After sterilizing the high-molecular-weight mixed solution with high pressure, it was combined with the drug-containing solution to obtain the DMN solution. The DMN solution was then applied onto a polydimethylsiloxane (PDMS) mold using vacuum suction to fill the holes with the solution. Finally, the DMNs were left to dry for more than 5 h in an environment with a relative humidity of not more than 30% (Figure 2).

### 2.4. Characterizations of DMNs

DMNs were fabricated by employing PDMS molds that were designed to have a needle height of 230 μm. We then evaluated the DMNs’ morphology and actual height by utilizing fluorescence microscopy (BX51, Olympus, Tokyo, Japan). Additionally, the dissolution characteristics of DMNs were investigated using detached piglet pig skin. DMNs were affixed to a hydrogel backing, and a microneedle applicator (20 N/cm${}^{2}$) was employed to exert pressure for 20 s, followed by a 40 min waiting period before the removal of DMNs. Finally, the needle height of the remaining DMNs was analyzed. According to a previous study [32], Parafilm sealing film was employed to evaluate the penetration performance of DMNs. DMNs were applied with a pressure of 20 N/cm${}^{2}$ for 20 s on an 8-layer sealing film. The DMNs were then removed to observe the puncture holes and maximum puncture depth formed on the sealing film. To further validate the puncture performance of DMNs, a needle injector (20 N/cm${}^{2}$) was used to facilitate the insertion of DMNs into excised porcine skin. Subsequently, the DMNs were removed and stained with a 4 mg/mL trypan blue dye solution for 30 min. Following the staining process, the dye was wiped off, and photographs were taken to document the array of needle punctures. Additionally, DMNs were also applied on the skin of SD rats, and the array formed on the rat’s skin was recorded.

### 2.5. The PTIO Antioxidant Activity of DMNs

The PTIO antioxidant test was adapted from a study by Li et al. [33]. Firstly, dissolve a single piece of DMN dry film in 1 mL of PBS buffer solution to obtain a 100% active solution. Next, prepare a solution with a concentration of 0.5 mg/mL each for PTIO and ASA. Distribute 1, 5, and 20 μL of the 100% active solution to the wells of a 96-well plate, adding PBS to all wells with less than 20 μL and setting up three replicates for each group. Use PBS as a blank control and ASA as a positive control. Finally, introduce 80 μL of PTIO solution into each well and incubate the plate in an incubator set at a constant temperature of 37 °C for 2 h. Next, complete processing by measuring absorbance at 570 nm using an enzyme-linked immunosorbent assay (ELISA) reader (Epoch2, BioTek). Compute the PTIO• scavenging rate using the formula:

(1) $$\mathrm{PTIO}•\;\mathrm{scavenging}\;\mathrm{rate}\%\;=\left(A_{0}-A\right)\times 100\%/A_{0}$$

where *A* and $A_{0}$ represent the absorbance values of the sample group and the control group, respectively.

### 2.6. DPPH• Scavenging Efficiency of DMNs

Antioxidant assays were performed according to the procedure described by Kim et al. [34], with appropriate modifications. A 100% DMN water solution was prepared as outlined in Section 2.5 and then diluted into concentrations of 40% and 10% with PBS solutions. Subsequently, 2 mg of DPPH powder was weighed and added to a 5 mL centrifuge tube, followed by the addition of 2 mL anhydrous ethanol to make a 1 mg/mL solution. The solution was sonicated in the dark for 5 min, shaken in the dark for 10 min, and then left undisturbed in the dark for 30 min until it was stable. The solution was further diluted to a concentration of 0.1 mg/mL. Positive control ASA was prepared in water to a concentration of 0.2 mg/mL. A 96-well plate was arranged with 100 μL of the different concentrations of the test solution, the positive control, and the negative control in each well. Each group was replicated three times. Then, 100 μL of DPPH solution was applied to each well. The plate was kept in the dark while it was agitated for 30 min. After this time, the absorbance was measured at 517 nm using an ELISA reader. The scavenging effect of DPPH radicals was measured using the formula:

(2) $$\mathrm{DPPH}•\;\mathrm{clearance}\;\mathrm{rate}\;\%=\left(A_{0}-A\right)\times 100\%/A_{0}$$

where *A* and $A_{0}$ represent the absorbance values of the sample group and the control group, respectively.

### 2.7. Safety on B16-F10 Cells

First, prepare the DMN extract. Sterilize the dry film with UV light for 24 h, then add a low-sugar DMEM culture medium in a ratio of one sheet per milliliter to obtain 100% extract. Next, prepare four concentration gradients of extract at 50%, 20%, 5%, and 1%. Seed B16-F10 cells in 96-well plates, with each well containing 8000 cells, and incubate for 24 h at 37 °C, 5% CO${}_{2}$, and saturated humidity in a culture box. Then, add 100 μL of different concentrations of DMN extract to each well, with four parallel groups set for each concentration and blank controls established at the same time. After administration, continue incubating for 24 h. After processing, wash the cells with PBS and add 100 μL of 10% Cell Counting Kit-8 (CCK-8) solution to each well, then continue incubation for 50 min. Finally, use an ELISA reader to measure the absorbance at 450 nm, and calculate cell vitality using the following formula:

(3) $$\mathrm{Cell}\;\mathrm{viability}\;\%=(A_{0}/A)\times 100\%$$

where *A* and $A_{0}$ represent the absorbance values of the sample group and the control group, respectively.

### 2.8. Effects of Cellular Tyrosinase Activity

The method developed by No et al. [35] was modified. Firstly, B16-F10 cells were seeded into 96-well plates at a density of 8000 cells/well and cultured for 48 h. The 100% concentration extraction solution was prepared following the method from Section 2.7. Then, it was diluted into three concentration gradients of 20%, 5%, and 1%. The test solution was added after 48 h, and each group was set up with 4 parallel experiments and a blank control. The cells were further incubated for 24 h after drug administration. After treatment, the cells were washed with PBS, and then 40 μL of cell lysis buffer containing 1 mM Phenylmethanesulfonyl fluoride was added to each well. The plate was incubated at 4 °C for 30 min, followed by incubation at 37 °C for 5 min. Subsequently, 100 μL of 1 mg/mL L-DOPA solution was added to each well and incubated in a 37 °C incubator for 2 h. After incubation, the activity of cellular tyrosinase was measured by obtaining the absorbance at 475 nm using an ELISA reader according to the following formula:

(4) $$\mathrm{Tyrosinase}\;\mathrm{activity}\;\%=(A_{0}/A)\times 100\%$$

where *A* and $A_{0}$ represent the absorbance values of the sample group and the control group, respectively.

### 2.9. Melanin Measurement

According to the methods described in a previous study [36], with appropriate modifications, B16-F10 cells were seeded at a density of 200,000 cells per well in a 6-well plate and incubated for 48 h. The extraction method for the solution was performed as described in Section 2.8. Following the incubation period, each well was treated with 2 mL of extraction solution at varying concentrations with a blank control and further incubated for 48 h. The cells were processed by washing with PBS twice and treated with 500 μL of cell lysis solution in each well, followed by incubation at room temperature for two hours for lysis. The melanin component was collected in a 1.5 mL centrifuge tube and centrifuged at a speed of 8000 rpm for 5 min. Then, the supernatant was discarded, and 200 μL of 1 M NaOH solution containing 10% DMSO was added to the samples. The samples were incubated in a 70–80 °C water bath for 4 h to dissolve the melanin. The solution was placed in a 96-well plate and the absorbance was measured at 405 nm using an ELISA reader. The melanin contents are calculated as follows:

(5) $$\mathrm{Melanin}\;\mathrm{contents}\;\%=(A_{0}/A)\times 100\%$$

where *A* and $A_{0}$ represent the absorbance values of the sample group and the control group, respectively.

### 2.10. Zebrafish Experiment

Fifteen zebrafish from the wild-type AB strain, exhibiting typical developmental characteristics after six hours of fertilization, were selected and assigned to each well of a six-well plate. Caution should be exercised while removing deionized water from the wells to avoid causing harm to the embryos. Following this, 3 mL of 0.5% DMN solution was rapidly added to each well, and a negative control group (adding 3 mL of deionized water) was established. The plate was wrapped in aluminum foil and incubated in a biochemical incubator at 28.5 °C for 45 h. Subsequently, ten zebrafish were randomly selected from each group for photography and observation. ImageJ software (version 1.53t) was employed to analyze the strength of the melanin signal present in their heads. Whitening efficacy was calculated using the formula below:

(6) $$\mathrm{Whitening}\;\mathrm{effect}\;\%=\left(S_{0}-S\right)\times 100\%/S_{0}$$

where *S* and $S_{0}$ represent the signal intensity of melanin in the head of the zebrafish in the sample group and the control group, respectively.

### 2.11. Clinical Research in DMNs

We recruited a total of 30 participants, consisting of 6 males and 24 females, with ages ranging from 20 to 58 years. Each participant applied DMNs for a duration of 24 h. At the conclusion of the application, the DMNs were removed, and the participants’ skin conditions were observed at 0.5, 24, and 48 h.

To study the clinical whitening efficacy of DMNs, six subjects were selected, two with facial freckles, three with melasma, and one with age spots. Prior to starting the trial, the 6 participants were required to undergo a standardized cleansing process. Following the cleansing process, the participants were instructed to sit in a room with standard conditions (temperature: 20–24 °C, humidity: 40–60%) for a minimum of 30 min. This was to ensure that the skin could naturally dry. Subsequently, a DMN patch was placed on the area of pigmentation beneath the right eye and left in place for one full night, a minimum of 8 h, before being removed. The DMN patch was applied every 48 h, totaling 28 applications. The use of any other products (such as cosmetics or topical medications) in the treated area was avoide during DMN application. The participants underwent facial imaging using a VISIA (a commercial skin imaging analyzer) before the trial and at weeks 2 (W2), 4 (W4), 6 (W6), and 8 (W8) to obtain pre- and post-treatment comparative data. The measured data comprised the water content of the stratum corneum at the application site, values of trans-epidermal water loss (TEWL), melanin, hematochrome, individual type angle (ITA), and a photograph of the subject’s facial area at the site of pigmentation. Tests on the same subject were completed by the same measurer.

### 2.12. Statistical Analysis

The quantitative data were analyzed through the IBM SPSS Statistics application, version 23.0, provided by IBM SPSS Inc. located in Chicago, IL, USA. Paired t-tests were used to analyze statistical differences in data that followed a normal distribution, either for within-group comparisons or for comparisons between the experimental and control groups. Significance levels of p<0.05, p<0.01, and p<0.001 are indicated by asterisks: *, **, and ***, respectively.

## 3. Results and Discussion

### 3.1. Preparation and Characterization of DMNs

**Figure 3:**
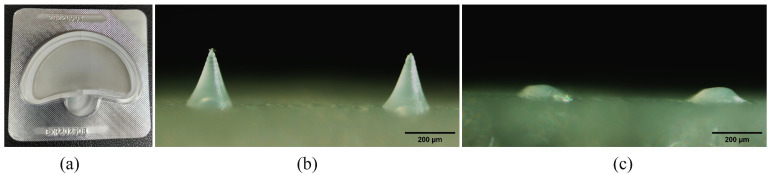
(**a**) DMNs prepared in a tens of thousands level clean room and packaged in aluminum and plastic; (**b**) DMN image captured after fabrication using a fluorescence microscope; (**c**) the needle tip that remains after inserting into pig skin and being dissolved in DMNs for 40 min.

**Figure 4:**
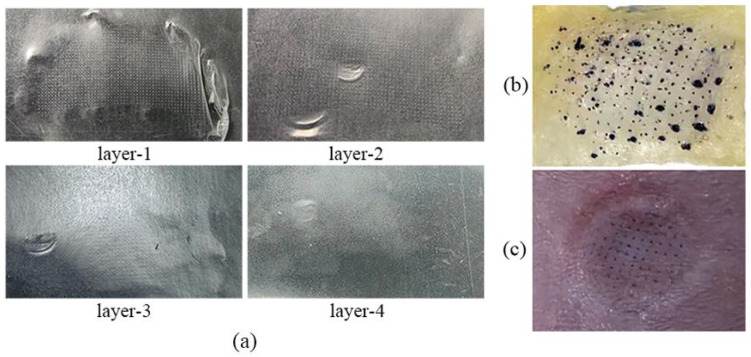
(**a**) Results of puncturing 1–4 layers of sealing film; the needle hole array obtained by applying DMNs to (**b**) pig skin and (**c**) the SD rat.

DMNs were manufactured in a clean room with a classification of ten thousand and packed with an aluminum–plastic foam cover, as illustrated in Figure 3a. A fluorescence microscope (BX51, Olympus, Tokyo, Japan) was used to capture images and observations, revealing that DMNs possess a complete needle tip structure. The needle height of the DMNs measures approximately 224.4 ± 4.4 μm (Figure 3b). In addition, after 40 min of skin (detached pigskin) treatment, the DMNs almost completely dissolved (Figure 3c). This indicates that DMNs have good dissolution ability, which can promote the rapid penetration of drugs into the skin. We conducted the puncture experiment for the DMNs using a sealant film with an average thickness of 126 μm per layer [32]. As illustrated in Figure 4a, the DMNs created a needle hole array on the first and second layers of the sealant film, but not on the third and fourth layers. Moreover, DMNs have the capability to create distinct arrays of needle punctures on both isolated pig skin and SD rat skin as illustrated in Figure 4b,c. These findings suggest that DMNs perform well in puncture tests.

The height of the MN tips ranges between 50 and 900 μm, allowing the MN to penetrate the stratum corneum [37]. However, an excessive needle height can cause skin damage. Xing et al. [38] assessed the impact of three various heights of MNs (230 μm, 500 μm, and 700 μm) on penetration depth and skin recovery. Among them, the depth of penetration of the 230 μm MNs into the skin was 85 ± 12 μm, and the skin could recover within 30 min. The stratum corneum has a thickness of approximately 10–20 μm. Consequently, a 230 μm needle can pierce through the stratum corneum, reaching the epidermis or shallow dermis, resulting in swiftly healing small wounds on the skin. As a result, we have selected a 230 μm needle height for the DMNs. Simultaneously, we selected HA, PVP, and PVA as matrix materials for the preparation of MNs. HA can hydrate and quickly absorb moisture, thereby accelerating the dissolution process of the DMNs [39]. The basal part of the DMNs, which is not inserted into the skin, will gradually dissolve and penetrate the skin through the pores created by the DMNs. Additionally, we utilized a PVA/PVP hybrid form to effectively address the lack of mechanical strength in PVA when used alone. PVA is widely used due to its excellent biocompatibility and ability to form films, yet its inability to provide sufficient mechanical strength can be solved by mixing with PVP [40].

### 3.2. Antioxidant Results of DMNs

**Figure 5:**
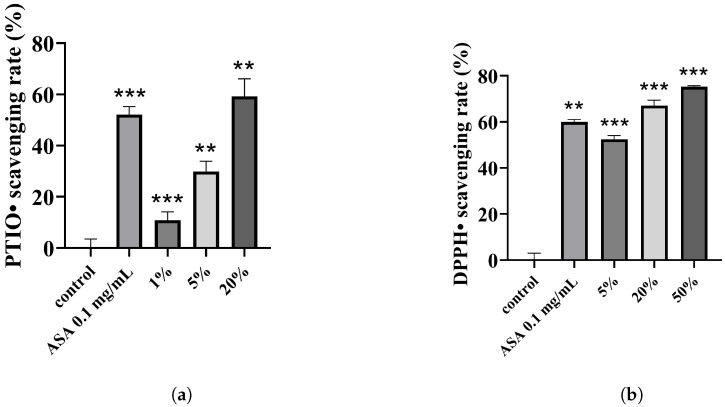
(**a**) PTIO• scavenging rate and (**b**) DPPH• scavenging rate of different concentrations of DMNs and positive control group (0.1 mg/mL ASA). Significance levels of *p* < 0.01, and *p* < 0.001 are indicated by asterisks: **, and ***, respectively.

We conducted two studies to assess the antioxidant capacity of the combination using the DPPH assay [41] and PTIO radical scavenging assay [42]. Results from the PTIO experiment indicate that the 5% DMN extract possesses modest antioxidant activity, with a PTIO• scavenging rate of approximately 30%, while the scavenging rate of the 20% extract surpasses that of the positive control (0.1 mg/mL ASA). Furthermore, the combination’s free radical scavenging efficacy positively correlates with the extract concentration (Figure 5a). Additionally, DMNs exhibit superior DPPH• clearing properties, with a 50% clearance rate at a 5% concentration and a higher clearance efficiency at a 20% concentration compared to the positive control (Figure 5b). Oxidative stress reactions arise from reactive oxygen species (ROS) accumulation [43] and may cause acquired melanin pigmentation. This can be countered by activating the cell’s antioxidant defense system [44] or by introducing antioxidants [45,46]. Therefore, we introduced the VC derivative EAA to the combination, and the experimental results confirm that the inclusion of EAA enhances the combination’s antioxidant effects.

### 3.3. Cytotoxicity Results of DMNs

**Figure 6:**
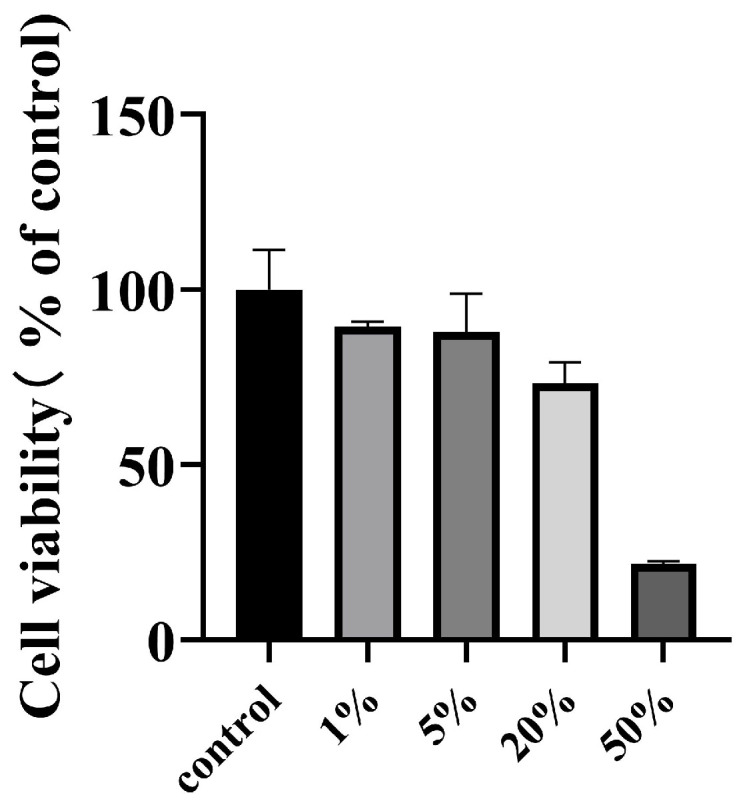
Cell viability of B16-F10 melanoma cells. Cells (8000) were seeded for 24 h, followed by treatment with different concentrations (1%, 5%, 20%, or 50%) of DMN extract for another 24 h. Untreated cells were used as controls.

The objective of this section is to evaluate the potential cytotoxicity of the DMN extract. We exposed the B16-F10 cells to different concentrations of DMN extract solution for 24 h. Then, we performed CCK-8 experiments to analyze their individual cytotoxic activities. The findings indicated that concentrations of 5% and below did not exhibit any cytotoxicity. Similarly, cellular viability at 20% concentration was approximately 75%, indicating non-cytotoxicity. In contrast, the 50% concentration showed higher cytotoxicity, as shown in Figure 6. This experiment provides a basis for our subsequent experiments on anti-melanogenesis and cellular tyrosinase activity.

### 3.4. Effect of DMNs on The Activity of Tyrosinase in B16-F10 Cells

**Figure 7:**
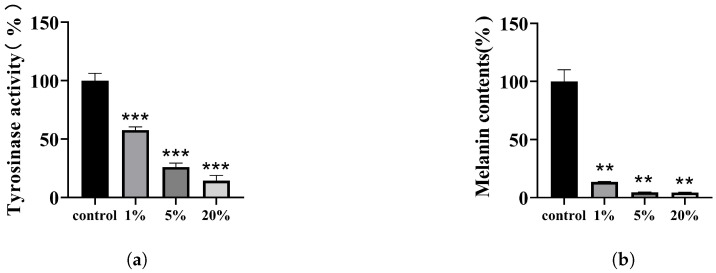
(**a**) Detection of tyrosinase activity in B16-F10 cells using L-DOPA. Cells were treated with various concentrations (1%, 5%, or 20%) of DMNs for 24 h. Controls were untreated. (**b**) Melanin contents of B16-F10 cells. Cells were treated with various concentrations of DMNs for 24 h. Controls were untreated. Significance levels of *p* < 0.01, and *p* < 0.001 are indicated by asterisks: **, and ***, respectively.

Tyrosinase is a crucial enzyme in the melanogenesis process [47]. To enhance the melanin inhibitory effect of the combination, we supplemented the tyrosinase inhibitor GLA. The tyrosinase inhibition effect of the combination was tested with B16-F10 cells. The outcomes demonstrated that at 1% concentration, about 42% of the enzyme activity was inhibited; at 5% concentration, around 74% was inhibited; and the inhibition rate at 20% concentration was over 80% (Figure 7a). It can be observed that the inhibition ability of the combination exhibited a direct correlation with the concentration. As a result, these findings suggest that DMNs have a greater tyrosinase inhibition efficiency.

### 3.5. Effect of DMNs on Melanin Production in B16-F10 Cells

For better visualization of the DMNs’ depigmentation effect, we quantitatively analyzed melanin using B16-F10 cells. As shown in Figure 7b, the DMNs’ effect on cellular melanogenesis was dose-dependent, inhibiting at rates of 86.43%, 95.35%, and 95.62% at concentrations of 1%, 5%, and 20% respectively. Previous tyrosinase experiments revealed that the tyrosinase inhibition rate for a 1% DMN extract was approximately 42%, whereas the melanin inhibition rate was 86%. Therefore, the combination of EAA, a potent antioxidant, and TXA, a melanin transfer inhibiting component, work synergistically with GLA to enhance its decolorization ability.

### 3.6. Effect of DMNs on Melanin Pigmentation of Zebrafish Embryos

Considering the prolonged duration and intricate procedures associated with guinea pig experiments, we therefore chose zebrafish as an animal model to evaluate the discoloration capacity of DMNs [48,49].

**Figure 8:**
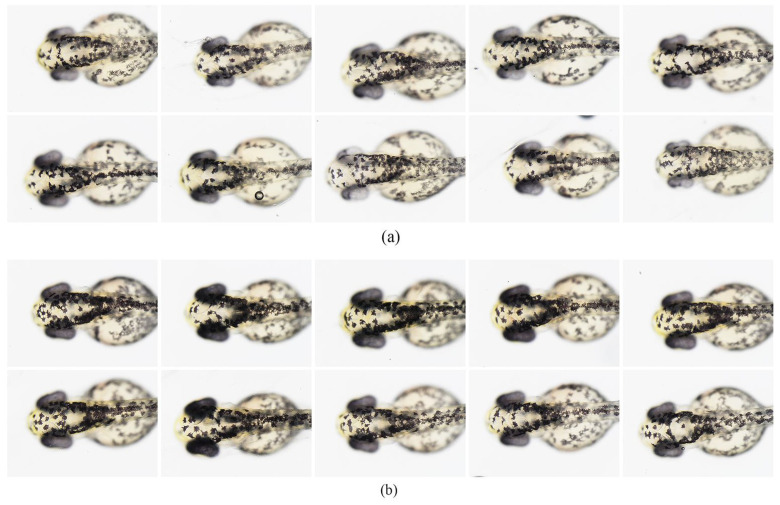
Representative photographs of zebrafish embryos at 51 h post fertilization (hpf). Embryos were treated with (**a**) 0.5% DMNs from 6 to 51 hpf; (**b**) Controls were untreated.

**Figure 9:**
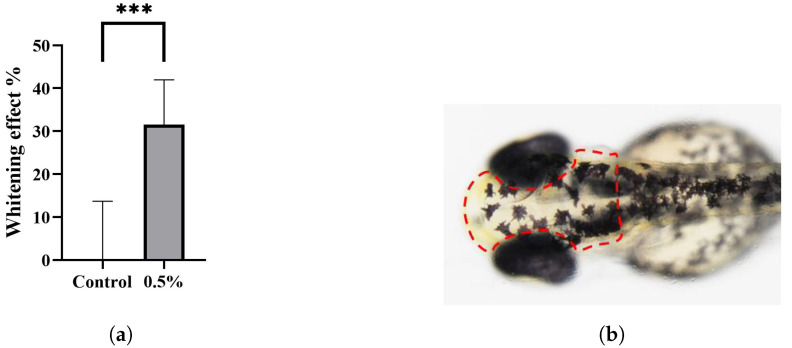
(**a**) Whitening efficacy evaluation of 0.5% DMNs and blank control. All groups were quantitatively analyzed for zebrafish head melanin using ImageJ software, and the results are expressed as mean ± SD (n = 10). (**b**) The melanin content is determined by analyzing the region enclosed by the red dashed box. The significance level of *p* < 0.001 is indicated by asterisk ***.

Ten zebrafish were selected randomly for each group to observe the back discoloration. The chromatophores on the backs of treated zebrafish were lighter than those in the control group (Figure 8a,b). Next, we used ImageJ software to evaluate the melanin on the backs of both groups of zebrafish quantitatively, and the decolorization rate in the treatment group rose to 32%, as shown in Figure 9a. The qualitative and quantitative results of the zebrafish experiment preliminarily verified that DMNs have decolorization potential. However, the depigmentation effect of the drug combination on zebrafish is relatively poor compared to its effects on B16-F10 cells. One possible reason is that the GLA in the combination does not exert a depigmentation effect on zebrafish and has a certain teratogenicity on zebrafish embryos [23]. The toxicity of GLA reduces the overall drug concentration of the combination. However, DMNs can achieve a depigmentation rate of 32% even at low administration concentrations and in the absence of GLA’s effectiveness, indicating DMNs’ whitening potential. In Figure 9b, the red dashed box delineates the region where we employed ImageJ software to quantify the concentration of melanin in the head of the zebrafish.

### 3.7. The Clinical Trial of DMNs

Thirty volunteers were recruited to assess the irritability of DMNs. Low-sensitivity adhesive tape was utilized to apply DMNs to the participants’ backs for 24 h, followed by removal. The participants’ skin reactions were observed at 0.5 h, 24 h, and 48 h, respectively. The results were recorded based on the skin reaction grading standards outlined in the Cosmetic Safety Technical Specifications (2015 version). The results indicated no occurrence of adverse reactions among these 30 volunteers, thus confirming the safety of DMN usage.

**Figure 10:**
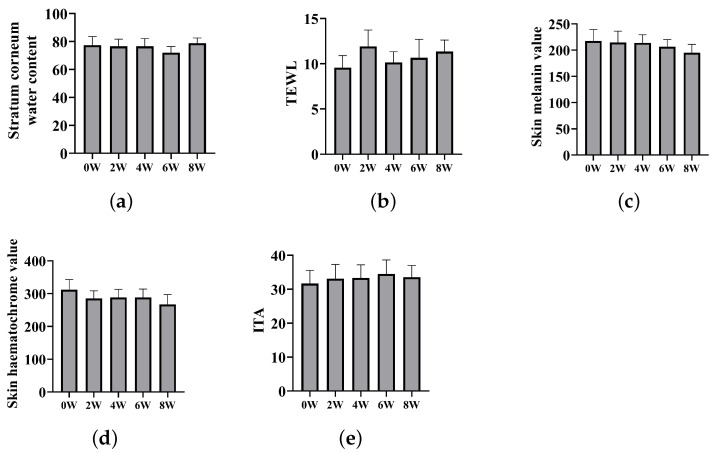
All subjects were tested at 0 W, 2 W, 4 W, 6 W, and 8 W for (**a**) skin stratum corneum water content, (**b**) TEWL, (**c**) skin melanin, (**d**) skin hematochrome, and (**e**) ITA.

**Figure 11:**
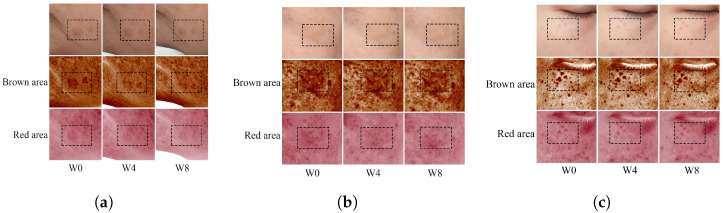
Subjects with (**a**) age spots, (**b**) melasma, and (**c**) freckles on the face were taken at 0 W, 4 W, and 8 W using VISIA (a commercial skin imaging analyzer) to capture facial images. The dashed box highlights the area of pigment reduction.

Due to its assured safety, we enlisted various volunteers exhibiting visible freckles, melasma, and age spots on their faces for an eight-week efficacy test of DMNs for skin whitening. During the procedure, there were no noticeable changes observed in the moisture content of the stratum corneum and the TEWL of all individuals (Figure 10a,b). This suggests that DMN application does not result in moisture loss in the stratum corneum. The primary component of DMNs, HA, exhibits potent hydrating properties and is extensively utilized in cosmetics to improve skin moisture retention [50], which might be the reason for there being no observed loss of moisture. The skin’s melanin and red pigment at the site of drug administration both decreased. Melanin decreased by 10.49% and red pigment decreased by 14.40% (Figure 10c,d). ITA (ITA is used to represent the chromatic individual type angle, with a higher value indicating a brighter skin color) value slightly increased by approximately 5.97% (Figure 10e), signifying that DMNs can reasonably lighten spots and whiten skin. We captured the participants’ facial images using VISIA (a commercial skin imaging analyzer) every four weeks. Patients with age spots exhibited a reduction in pigmentation in the brown and red regions (Figure 11a), while patients with melasma showed slight a reduction in the pigment in their faces (Figure 11b), but it was less than that of age spots. Nonetheless, the treatment effect of DMNs on freckles was not significant (Figure 11c). The reason for the different efficacies of DMNs on melasma, age spots, and freckles may be due to the reality that freckles are primarily determined by genetic and congenital factors, whereas melasma and age spots are primarily caused by postnatal factors such as UV exposure and hormone levels [51]. Meanwhile, most drugs are more effective in treating postnatal diseases rather than congenital diseases. In conclusion, clinical trials have validated the safety of DMNs for topical application and have shown that it does not cause any loss of skin moisture. Moreover, DMNs exhibit selective efficacy in treating both age spots and melasma.

## 4. Conclusions

UV radiation triggers oxidative stress, which leads to an increase in intracellular ROS levels in human epidermal melanocytes [52]. Additionally, UV exposure increases the activity of intracellular tyrosinase, accelerating melanin production in the epidermis [53]. In this study, a polymer matrix containing GLA, EAA, and TXA was loaded onto MNs, and the antioxidant effects of DMNs were confirmed. DMNs inhibit intracellular tyrosinase and melanin production, as demonstrated in B16-F10 cells and zebrafish models. DMNs were found safe in clinical trials and selectively lightened chloasma and age spots. Future research will determine the optimal ratios of the three-drug components. In conclusion, the DMNs, which consist of various components and exhibit multiple effects, have the potential for whitening. 

## Data Availability

Data are available on request due to restrictions, e.g., privacy or ethics; the data presented in this study are available on request from the corresponding author.

## Abbreviations

MNs, microneedles; DMNs, drug-loaded microneedles; ASA, ascorbic acid; TXA, Tranexamic acid; GLA, Glabridin; EAA, 3-O-Ethyl-L-ascorbic acid; VC, vitamin C; HA, hyaluronic acid; PVA, Poly(vinyl alcohol); PVP, poly(vinylpyrrolidone); PDMS, polydimethylsiloxane; ELISA, enzymelinked immunosorbent assay; CCK-8, Cell Counting Kit-8; TEWL, trans-epidermal water loss; ITA, individual type angle; ROS, reactive oxygen species; PTIO, 2-Phenyl-4,4,5,5-tetramethylimidazoline-1-oxyl-3-oxide; DPPH, 1,1-diphenyl-2-picrylhydrazyl.
